# Machine Learning Models for Predicting Thermal Properties of Radiative Cooling Aerogels

**DOI:** 10.3390/gels11010070

**Published:** 2025-01-16

**Authors:** Chengce Yuan, Yimin Shi, Zhichen Ba, Daxin Liang, Jing Wang, Xiaorui Liu, Yabei Xu, Junreng Liu, Hongbo Xu

**Affiliations:** 1 AVIC Shenyang Aircraft Corporation, Shenyang 110850, China; yuanc3100@163.com; 2 Key Laboratory of Bio-Based Material Science and Technology (Ministry of Education), Northeast Forestry University, Harbin 150040, China; 3 School of Chemistry and Chemical Engineering, Harbin Institute of Technology, Harbin 150001, China; iamxhb@hit.edu.cn

**Keywords:** radiative cooling aerogels, machine learning, SHAP analysis

## Abstract

The escalating global climate crisis and energy challenges have made the development of efficient radiative cooling materials increasingly urgent. This study presents a machine-learning-based model for predicting the performance of radiative cooling aerogels (RCAs). The model integrated multiple parameters, including the material composition (matrix material type and proportions), modification design (modifier type and content), optical properties (solar reflectance and infrared emissivity), and environmental factors (solar irradiance and ambient temperature) to achieve accurate cooling performance predictions. A comparative analysis of various machine learning algorithms revealed that an optimized XGBoost model demonstrated superior predictive performance, achieving an R^2^ value of 0.943 and an RMSE of 1.423 for the test dataset. An interpretability analysis using Shapley additive explanations (SHAPs) identified a ZnO modifier (SHAP value, 1.523) and environmental parameters (ambient temperature, 1.299; solar irradiance, 0.979) as the most significant determinants of cooling performance. A feature interaction analysis further elucidated the complex interplay between the material composition and environmental conditions, providing theoretical guidance for material optimization.

## 1. Introduction

Global climate change and the energy crisis have intensified, with building energy consumption emerging as a major component of total energy use. Air-conditioning systems account for over 40% of building energy consumption, a proportion that continues to rise. Traditional vapor-compression cooling technologies not only consume substantial energy but also generate significant greenhouse gas emissions, severely impacting environmental sustainability [1]. Consequently, developing new energy-efficient and environmentally friendly cooling technologies has become imperative.

Aerogels have demonstrated unprecedented potential in radiative cooling applications [2,3,4] due to their exceptional thermal insulation properties (thermal conductivity below 0.02 W/m·K), ultra-high porosity (exceeding 90%), and highly controllable optical characteristics. These unique physical properties enable aerogels to effectively reflect or emit infrared radiation, thereby reducing heat absorption and achieving cooling effects. Through the precise adjustment of the chemical composition [5], microstructure (including pore size and distribution), and surface morphology [6], researchers can now achieve precise control over material radiative properties without compromising mechanical strength [7]. For instance, Cai’s research group [8], inspired by the structural whiteness of butterfly wings, developed a sustainable cellulose nanocrystal aerogel with gratings designed for efficient radiative cooling.

However, despite their promising applications in radiative cooling, the complexity of aerogel preparation remains a significant challenge [9]. Aerogel synthesis involves multiple steps, including sol–gel transformation, aging, and drying processes, each potentially affecting the final product performance. The presence of multiple variables makes traditional trial-and-error optimization methods both inefficient and costly. Moreover, while understanding and accurately predicting the physicochemical behavior of aerogels is crucial for advancing the field, conventional experimental testing and theoretical modeling approaches typically require substantial time [10] and resource investments, significantly increasing research and development challenges.

Machine learning, a crucial branch of artificial intelligence, has achieved significant breakthroughs in materials science in recent years. By establishing data-driven models of material structure-property relationships, machine learning has enabled the rapid prediction of material properties, substantially reducing development cycles. Previous research has demonstrated successful applications in energy and optical materials. For instance, Ding et al. [11] integrated machine learning into the design of Batocera-Lineolata-Hope-inspired photonic structures using SiO_2_ as the base material. Their deep learning model comprehended complex relationships between biomimetic metamaterials and spectral responses, achieving a high average emissivity of 0.985 in the 0.8–2.4 range. Similarly, Guang et al. [12] employed a hybrid integer programming algorithm for design optimization, simultaneously optimizing layer materials, sequence numbering, and thickness while utilizing cascaded neural networks for on-demand color generation using bottom nanocavity structure determination. This machine-learning-assisted inverse design method enabled real-time structural predictions for on-demand color generation. However, the application of machine learning to predict RCA performance remains unexplored, presenting a significant research opportunity.

**Figure 1:**
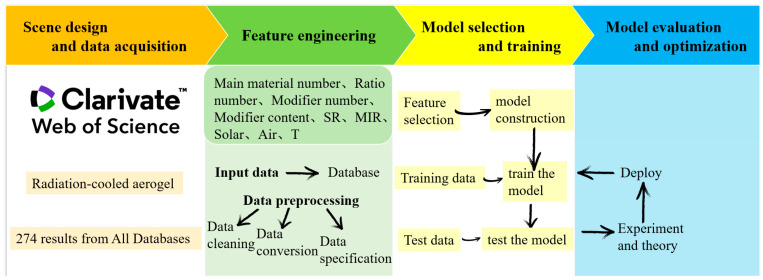
Preparation of the datasets used to train the machine learning model.

Addressing this gap, our study developed a machine-learning-based model for predicting radiative cooling aerogel performance. As shown in Figure 1, the model innovatively incorporated multidimensional features as input variables, including material composition parameters (matrix material type and proportions), modification design parameters (modifier type and content), optical performance parameters (solar reflectance and infrared emissivity), and environmental factors (solar irradiance and ambient temperature). Unlike previous studies limited to single material systems, this model systematically considered the impact of modification particles with superior mid-infrared emissivity on aerogel performance. Importantly, by incorporating environmental parameters, the model could flexibly predict actual cooling effects under various climatic conditions, providing valuable insights for practical applications. This novel ’forward-prediction–inverse-design’ strategy not only significantly enhances the development efficiency of radiative cooling aerogels but also establishes a new research paradigm for the intelligent design of other functional materials.

## 2. Results and Discussion

### 2.1. Statistical Analysis of Pre-Modeling Data

**Figure 2:**
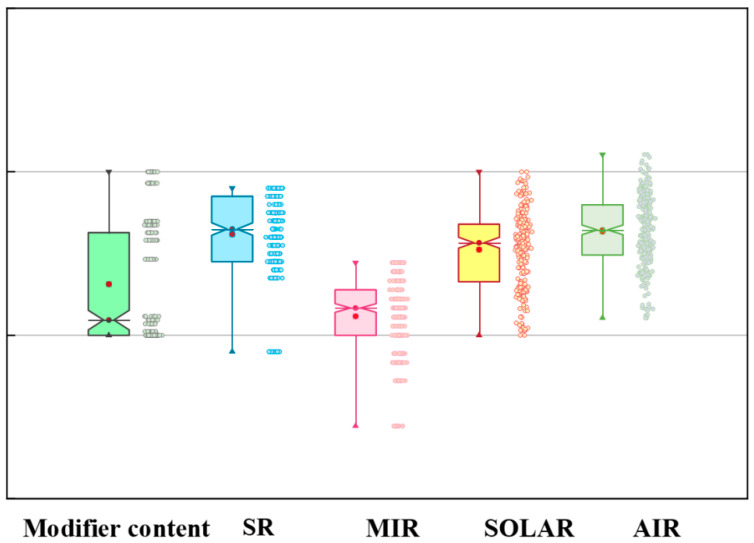
Statistics of the dataset. Modifier content (green box), SR (blue box), MIR (pink box), SOLAR (yellow box), and AIR (light green box).

Based on a systematic literature review and data collection, we established a dataset encompassing 31 variables. The descriptive analysis of the collected data employed one-hot encoding for the matrix material types and modification material types, while the modifier content, solar reflectance, infrared emissivity, solar irradiance, and ambient temperature were represented using quartile-based box plots (Figure 2). Each box plot comprised the following five numerical points: minimum observation (lower whisker), 25%, median, 75%, and maximum observation (upper whisker). For detailed numerical ranges and a complete statistical analysis of all features, please refer to Table S1 in the Supplementary Materials.

These variables were selected based on fundamental radiative cooling principles and established material property relationships. The matrix material types and their ratios determine the basic structure and porosity of aerogels. Modifier types and content directly influence the optical properties and thermal performance. Environmental parameters (solar irradiance and ambient temperature) are essential for evaluating actual cooling performance under different conditions. The optical properties (solar reflectance and infrared emissivity) represent the key mechanisms of radiative cooling materials.

This study encompassed a comprehensive range of aerogel materials, systematically categorized into three main classes based on their chemical composition and structural characteristics. The first category consisted of natural polymer-based aerogels, including cellulose nanofiber (CNF) and chitosan (CS), which are derived from renewable resources. The second category comprised synthetic polymer-based aerogels, featuring polyacrylamide (PAAm), polydimethylsiloxane (PDMS), polyester amide (PEA), polylactic acid (PLA), polypropylene urethane (PPU), polyvinyl alcohol (PVA), polyvinylidene fluoride (PVDF), and sodium alginate (SA). The third category included inorganic aerogels, specifically, calcium silicate (CaSiO_3_) and methyltrimethoxysilane (MTMS).

A statistical analysis revealed an average modifier content of 13.493%. This relatively low proportion plays a crucial role in radiative cooling aerogels, significantly affecting both infrared emissivity and overall cooling performance. The modifier content must be carefully balanced [13]; insufficient amounts may compromise radiative cooling performance, while excessive amounts could disrupt the aerogel’s porous structure and thermal conductivity properties. This statistical finding provides valuable guidance for material composition optimization and performance control in practical applications.

The samples demonstrated an average solar reflectance of 0.924, indicating an excellent solar radiation reflection capability, crucial for minimizing daytime heat absorption. The standard deviation of 4.54 suggests that precise control over solar reflectance properties can be achieved through various material design and modification strategies [14,15] within a narrow range. The infrared emissivity averaged 0.921, with a standard deviation of 4.21, demonstrating stable high-emission characteristics in the atmospheric window (8–13 μm) range. This high infrared emissivity ensures efficient heat-radiation release, a key indicator for passive radiative cooling.

Notably, our study incorporated test data across diverse environmental conditions. The ambient temperature range spanned 5–50 °C, with a mean of 32.3 °C, encompassing various seasonal and geographical application scenarios. Solar irradiance measurements ranged from 0 to 1000 W/m^2^, with an average of 477.59 W/m^2^, covering various lighting conditions from overcast to clear skies and from dawn to noon. These comprehensive environmental data enabled the accurate prediction of material cooling performance under diverse climatic conditions, overcoming the limitations of testing under idealized conditions alone.

### 2.2. Analysis of Input Features Prior to Model Development

**Figure 3:**
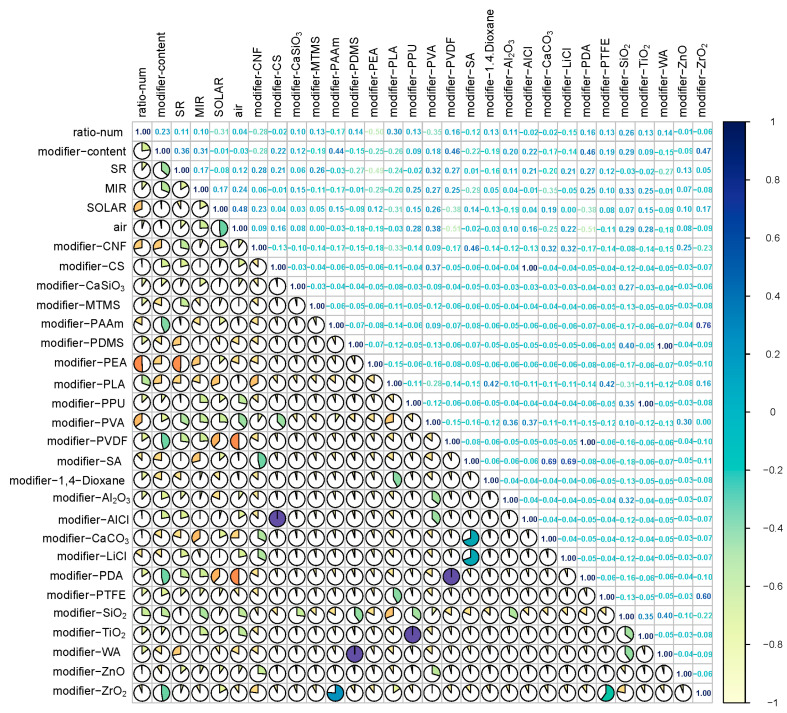
Pearson correlation matrix between any two features.

A Pearson correlation analysis was employed to assess the strength and direction of linear relationships between variables, enabling the determination of feature correlations. The Pearson correlation coefficients [16] were used to evaluate the strength of linear associations among different variables. Figure 3 presents the correlation coefficient matrix for all input features, with coefficients ranging from −1 to 1. The color gradient transitioned from light-yellow to deep-blue, representing the progression from negative to positive correlations.

The analysis results indicated that most features exhibited weak correlations (|r| < 0.3), confirming the independence of selected features and supporting accurate model predictions. Among the material parameters, ratio_num showed a moderate positive correlation with modifier_content (r = 0.23), reflecting an inherent relationship between material proportions and modifier content. Regarding the optical performance parameters, solar reflectance (SE) demonstrated a weak positive correlation with infrared emissivity (MIR) (r = 0.17) [9], suggesting these key performance indicators could independently be controlled through material design. The environmental factor analysis revealed a significant positive correlation between solar irradiance (SOLAR) and ambient temperature (air) (r = 0.48), aligning with established physical principles. Notably, certain modifiers exhibited strong correlations, such as modifier_CS with modifier_PVDF (r = 1.00) and modifier_PAAm with ZrO_2_ (r = 0.76) [17], providing valuable insights for material formulation optimization. The weak correlations observed between the environmental parameters and material characteristics indicated the good environmental adaptability of the studied aerogels.

### 2.3. Model Development and Optimization

**Figure 4:**
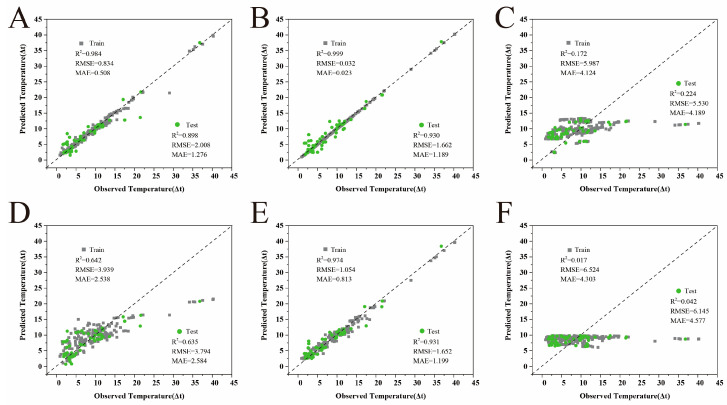
Regression plots of true vs. predicted values of RCAs using (**A**) RF, (**B**) XGBoost, (**C**) lasso, (**D**) MLP, (**E**) GBR, and (**F**) SVM models. The gray dotted lines show the perfect regression line (i.e., y = x).

To accurately predict the cooling performance of RCAs, six machine learning models (Figure 4) were developed, comprising three linear models (support vector machine, lasso regression, and multi-layer perceptron) and three nonlinear models (random forest, gradient-boosting regression, and XGBoost). Model performance was evaluated by comparing the models’ predictions for both training and test sets. The predictive capabilities for RCA cooling performance were assessed using selected input features across different models. Regression plots were employed to visualize the RMSE and MAE for the selected models. Superior predictive performance [18] was indicated by higher test R^2^ values and lower RMSE and MAE values.

An overall performance analysis revealed that the tree-based ensemble [19] nonlinear models demonstrated significant advantages. The XGBoost model achieved optimal predictive performance, with an R^2^ value of 0.930 on the test set and RMSE and MAE values of 1.662 and 1.189, respectively. This indicated the model’s ability to accurately capture complex nonlinear relationships between material characteristics and cooling performance. Notably, XGBoost achieved an R^2^ value of 0.9999 and an RMSE of just 0.032 on the training set, demonstrating an excellent fitting capability. The GBR and RF models followed closely, with test-set R^2^ values of 0.931 and 0.898, respectively. All three nonlinear models exhibited strong generalization performance.

In contrast, the linear models showed relatively inferior predictive performance. The traditional SVM model performed poorest, achieving an R^2^ value of only 0.042 and an RMSE of 6.145 on the test set. The lasso regression performed marginally better but still only achieved an R^2^ value of 0.224 and an RMSE of 5.530 on the test set. These results confirmed significant nonlinear relationships between radiative cooling aerogel performance and material characteristics. Although MLP can theoretically fit arbitrary nonlinear functions, its test-set performance (R^2^ = 0.635; RMSE = 3.794) remained significantly below the tree ensemble models, possibly due to model structure optimization and parameter-tuning considerations.

From a robustness perspective, the nonlinear models demonstrated superior stability. The XGBoost and GBR models showed relatively small performance disparities between the training and test sets, indicating strong generalization capabilities [20] and resistance to overfitting. The GBR model particularly excelled in this aspect, showing the smallest RMSE difference between the training and test sets (1.054 and 1.652, respectively), demonstrating stable predictive capabilities.

**Figure 5:**
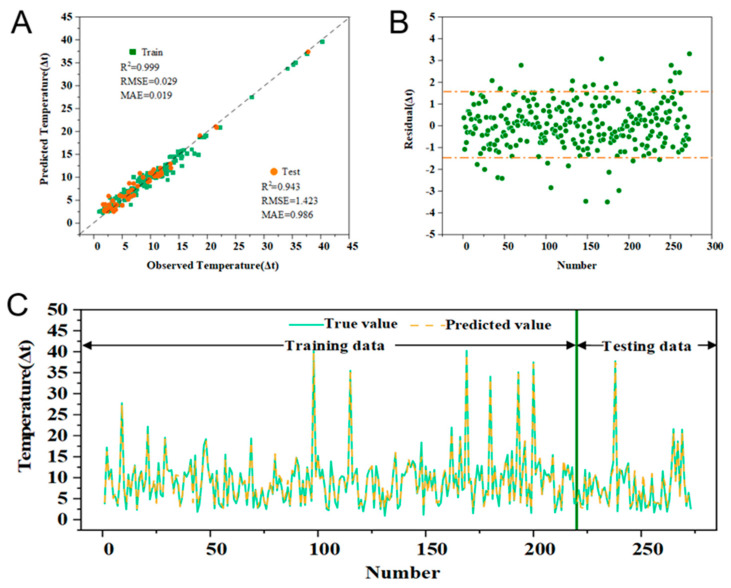
(**A**) Hyperparametric optimized XGBoost model. (**B**) Residual distribution of predicted results. (**C**) Distribution of true and predicted values for the datasets (training data and testing data).

To further enhance model performance, the XGBoost model was optimized using the Optuna framework [21]. Optuna employs Bayesian optimization algorithms to efficiently search predefined parameter spaces, primarily optimizing hyperparameters including maximum tree depth (max_depth) and minimum child weight (min_child_weight). The optimization process utilized a 5-fold cross-validation with the mean R^2^ value as the optimization objective. The optimized XGBoost model demonstrated superior performance on both training and test datasets (Figure 5A) [22], significantly surpassing the original model’s capabilities. The x-axis showed the sample index number, representing the sequential ordering of the samples in our dataset.

Specifically, the optimized model achieved improved R^2^ values of 0.999 and 0.943 on the training and test sets, respectively, while the RMSE values decreased to 0.029 and 1.423 and the MAE values reduced to 0.019 and 0.986. Figure 5B illustrates the residual distribution between the predicted and actual values, with predictions on the x-axis and residuals (actual minus predicted values) on the y-axis. The residual plot revealed a relatively random distribution pattern [23], with most residuals concentrated within ±1.5 °C and uniformly distributed around the zero line. This distribution pattern indicated the absence of systematic bias, with prediction errors showing no significant trends relative to the predicted values.

An uncertainty analysis, as shown in Figure 5C, demonstrated reasonable and stable prediction intervals, with the majority of observations falling within the 95% confidence interval, validating the model’s reliability. Furthermore, the optimized XGBoost model achieved a higher prediction interval coverage probability (PICP) for both the training and test sets, further confirming the model’s accuracy and stability. Based on these comprehensive performance evaluations, the optimized XGBoost model exhibited exceptional predictive capability and stability, warranting its selection as the final prediction model for this study.

### 2.4. Feature Importance and Interpretability Analysis

**Figure 6:**
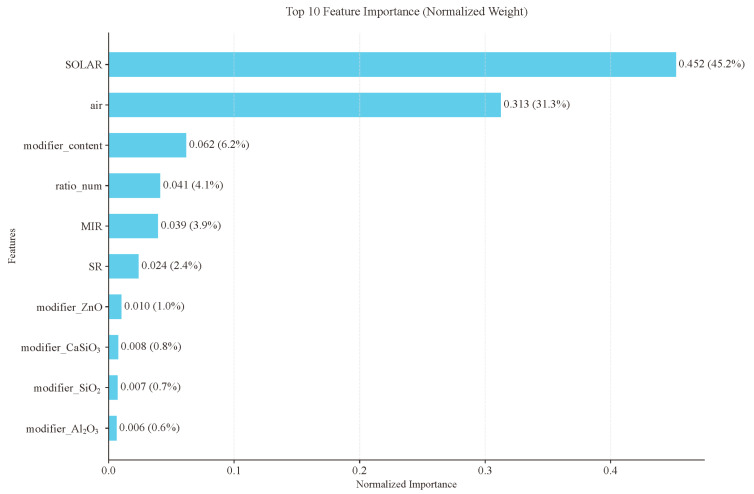
Feature importance results from XGBoost model.

Feature importance is a quantitative metric in machine learning models that evaluates the impact of each input feature on prediction outcomes. Higher importance scores indicate [24] greater influence of a feature on model predictions. To gain a deeper insight into the key factors affecting radiative cooling aerogel performance, we conducted a feature importance analysis using the optimized XGBoost model (Figure 6). By calculating each feature’s contribution to model predictions [25], we quantified the influence of different parameters on cooling performance, providing theoretical guidance for material design.

The feature importance analysis revealed that environmental factors played a dominant role in determining cooling performance. SOLAR demonstrated the highest normalized importance at 0.452, accounting for 45.2% of the total importance, indicating that incident solar radiation intensity is the most critical factor [26] affecting cooling efficiency. This was followed by air temperature, with an importance of 0.313 (31.3%). Together, these environmental parameters contributed 76.5% of the total feature importance, emphasizing the decisive influence of environmental conditions on radiative cooling performance.

Among the material design parameters, modifier content ranked third at 6.2% importance, indicating its significant influence on material performance modulation. The material composition ratio (ratio_num) and MIR contributed 4.1% and 3.9% importance, respectively, suggesting that the matrix material composition and infrared radiation characteristics [27] were also substantial factors. Solar reflectance (SE) showed 2.4% importance, which, although relatively small, remains significant for optimizing daytime cooling efficiency.

To enhance model interpretability, we employed a SHAP (Shapley additive explanation) [28] analysis on the XGBoost model. SHAP values calculate the marginal contribution of each feature to model predictions using game theory principles, providing both an overall feature importance assessment and a specific prediction impact interpretation.

**Figure 7:**
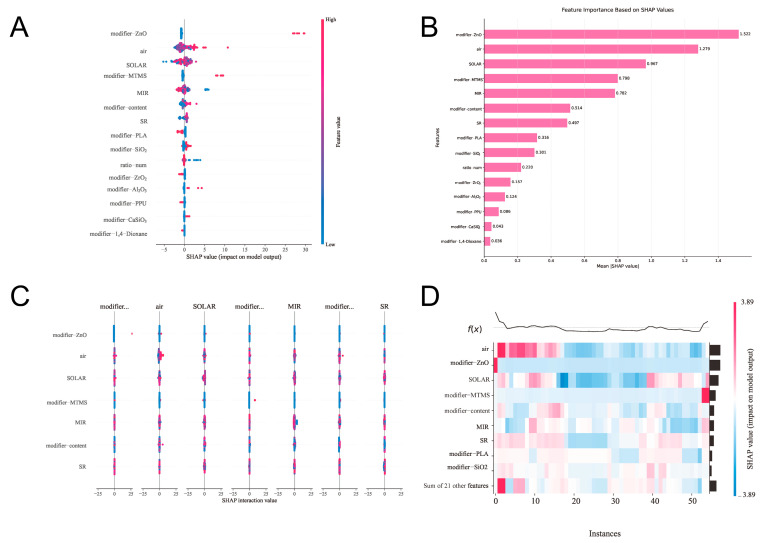
SHAP-based feature importance. (**A**) Summary plot of SHAP. (**B**) Factor importance plot. (**C**) Summary plot of SHAP between two features. (**D**) Feature impact heat map.

Figure 7A presents global feature importance, with the ZnO modifier showing the highest average SHAP value (1.523), indicating its predominant influence on cooling performance. This could be attributed to ZnO’s unique optical and structural characteristics [29]. Its high emissivity in the mid-infrared region (8–13 μm) enhances aerogel thermal radiation in the atmospheric window band, while the incorporation of ZnO nanoparticles increases solar radiation reflection through scattering and reflection mechanisms while maintaining the aerogel’s porous structure, thereby reducing solar energy absorption. This finding complemented the previous feature importance analysis, revealing ZnO’s crucial role at the microscopic level. Environmental parameters (air and SOLAR) ranked second and third, with SHAP values of 1.299 and 0.979, respectively, reconfirming the significance of environmental conditions.

Figure 7B clearly illustrates the manner and degree of each feature’s impact on the model output. ZnO’s SHAP values showed the widest distribution, with notable outliers in the positive range (particularly between 25 and 30), indicating significant cooling performance enhancement under specific conditions. MIR demonstrated a more concentrated distribution with bidirectional extensions, reflecting its dual regulatory effect on cooling performance.

Although the correlation analysis in Section 2.2 showed relatively weak linear correlations for ZnO, the SHAP analysis revealed its significant nonlinear impact on cooling performance. This apparent discrepancy highlighted the limitation of the linear correlation analysis at capturing complex structure-property relationships. ZnO’s high SHAP value (1.523) indicated that its influence on cooling performance involved sophisticated mechanisms and interactions with other material parameters, which could not be captured by a simple linear correlation analysis. This finding demonstrates the advantage of using advanced machine learning techniques and SHAP analyses to uncover complex material property relationships that might be overlooked by traditional statistical methods.

From the SHAP analysis, we found that the choice of matrix materials significantly impacted cooling performance. Matrix materials such as CS, PVDF, and PAAm, due to their unique molecular structures and optical properties, demonstrated varying degrees of interaction with the modifier. Particularly, when the matrix material ratio (ratio_num) was optimized, it could better enhance the performance modulation effect of the modifier.

A further SHAP analysis revealed distinct performance contributions from different modifiers. ZnO demonstrated superior performance (SHAP value, 1.523), primarily due to its unique optical properties in the mid-infrared region (8–13 μm) and high refractive index. TiO_2_ showed the second-highest impact (SHAP value, 0.856), attributed to its excellent solar reflection capabilities. Al_2_O_3_ (SHAP value, 0.624) exhibited a moderate influence, mainly through its thermal radiation properties. The interaction analysis between modifier types and content revealed optimal performance windows: ZnO achieved peak effectiveness at 10–15 wt%, while TiO_2_ and Al_2_O_3_ showed optimal performance at slightly different concentrations (8–12 wt% and 12–18 wt%, respectively). This variance in optimal content ranges reflected the distinct working mechanisms of different modifiers in enhancing radiative cooling performance.

## 3. Conclusions

This study established a machine-learning-based model for predicting radiative cooling aerogel performance through systematic data collection, feature engineering, and model optimization. A comparative analysis of six machine learning models revealed that the optimized XGBoost model demonstrated superior predictive performance. After Optuna hyperparameter optimization, the model achieved an R^2^ value of 0.943 and an RMSE of 1.423 on the test set, confirming its effectiveness and reliability in predicting radiative cooling performance. An interpretability analysis using the SHAP method revealed key factors influencing cooling performance. Notably, the ZnO modifier (SHAP value, 1.523) played a decisive role in enhancing material cooling performance through its unique optical and structural characteristics. The significant impact of environmental parameters (ambient temperature and solar irradiance) indicated that the material design must thoroughly consider the environmental characteristics of the application scenario. A feature interaction analysis uncovered complex synergistic effects between material components and environmental conditions, providing crucial guidance for multi-component modification strategies. In particular, optimizing the modifier content and balancing environmental adaptability proved critical for enhancing material performance. The predictive model developed in this study not only enabled a rapid performance assessment but also guided material optimization through a reverse analysis, offering new research directions for developing high-performance radiative cooling aerogels. This machine-learning-integrated approach to material design significantly improves material development efficiency.

The innovations of this study lie not only in the high precision of the predictive model but also in establishing a systematic methodology from data-driven approaches to material design, providing a paradigm for developing novel functional materials. This research strategy, combining materials science with artificial intelligence, demonstrates broad application prospects.

## 4. Materials and Methods

### 4.1. Data Collection and Preprocessing

This study utilized Web of Science as the primary data source, employing ‘radiative cooling’ and ‘aerogel’ as search keywords to collect 274 experimental data points. The dataset encompassed the preparation conditions, component ratios, modification methods, and performance characteristics of various radiative cooling aerogel materials. To ensure data quality, rigorous screening and standardization procedures were implemented, including the removal of duplicate entries, treatment of missing values, elimination of outliers, and normalization of features with different dimensions to the [0, 1] interval. Categorical features, such as matrix material types and modifier varieties, were converted to binary numerical features using one-hot encoding [30] to avoid introducing artificial ordinal relationships. Other categorical features, such as material types, were transformed into numerical features using appropriate encoding methods. The final dataset incorporated features including the matrix material type, material composition ratios, modifier types, modifier content, solar reflectance, infrared emissivity, solar irradiance, and ambient temperature, with cooling temperature reduction as the target variable.

The final dataset incorporated the following features: matrix material type (using one-hot encoding for materials such as CNF, CS, PVDF, etc.); material composition ratios (ratio_num); modifier types (using one-hot encoding for modifiers such as ZnO, TiO_2_, Al_2_O_3_, etc.); modifier content (modifier_content, wt%); optical properties, including solar reflectance (SR, dimensionless) and infrared emissivity (MIR, dimensionless); and environmental parameters, including solar irradiance (SOLAR, W/m^2^) and ambient temperature (air, °C), with cooling temperature reduction (ΔT, °C) as the target variable.

For detailed information about the data sources [2,4,8,15,31,32,33,34,35,36,37,38,39,40,41,42,43,44,45,46], readers are referred to Data S2 in the Supplementary Materials.

### 4.2. ML Model Development

Based on the optimized RCA database, the dataset was split into training and test sets at an 80:20 ratio to evaluate model generalization performance. The study compared three linear models (support vector regression (SVM), lasso regression, and multi-layer perceptron (MLP)) and three nonlinear models (random forest (RF), gradient-boosting regression (GBR), and XGBoost). The linear models were selected for their simplicity and interpretability [47] for material property prediction, while the nonlinear models were chosen for their superior capability at capturing complex nonlinear relationships between material characteristics and performance. Tree-based ensemble algorithms (RF, GBR, and XGBoost) particularly excel at handling high-dimensional features and complex interaction effects.

After model training, validation was performed using the test set, comparing the simulated results with actual outcomes to verify the accuracy of the different models. Model predictive performance was quantified using key statistical parameters, including root mean square error (RMSE), mean absolute error (MAE), and coefficient of determination (R^2^). RMSE measures the deviation between predicted and actual values, reflecting prediction accuracy. MAE serves as an intuitive indicator of model errors, with values approaching zero indicating higher accuracy. R^2^ indicates the degree of fit between model estimates and observed values, with values approaching 1 demonstrating superior performance. The RMSE, MAE, and R^2^ [48] were calculated using Equations (1)–(3) as follows:

(1) $$RMSE=\sqrt{\frac{\sum_{i=1}^{n}{\left(y_{t}-y_{p}\right)}^{2}}{n}}$$

(2) $$MAE=\frac{\sum_{i=1}^{n}\left|y_{t}-y_{p}\right|}{n}\;$$

(3) $$R^{2}=1-\frac{\sum_{i=1}^{n}{\left(y_{p}-y_{t}\right)}^{2}}{\sum_{i=1}^{n}{\left(y_{t}-y_{m}\right)}^{2}}\;$$

where *y_p_* represents the predicted output value, *y_t_* denotes the reported true output value, *y_m_* represents the mean of the observed output values, and n indicates the number of samples in the training or testing datasets.

### 4.3. Model Optimization and Interpretation

After training and testing six different models, the most accurate model was selected for optimization. The Optuna hyperparameter optimization framework [21] was employed to determine the optimal hyperparameters, followed by model fine-tuning to further enhance prediction accuracy. Model interpretation was conducted using multiple complementary approaches. A feature importance analysis was performed using the random forest model’s feature importance ranking function to evaluate the significance of factors affecting both enthalpy and phase-change temperature, where higher importance values indicated greater feature impact on target variables. The SHAP method was implemented to calculate marginal feature contributions to model output, providing both global and local explanations of the black-box model and generating SHAP values [49] for each predicted sample to quantify descriptor influence on individual data points. Additionally, partial dependence plots (PDPs) [50] were utilized to visualize relationships between individual features and output variables, examine feature set interactions while marginalizing complementary features, and demonstrate the positive and negative effects of input variables on model predictions.

The machine learning methodology developed in this study demonstrates strong potential for broader applications in aerogel research. The same approach of combining feature engineering, XGBoost modeling, and SHAP analyses could be adapted to predict and optimize other crucial aerogel properties such as mechanical strength, thermal conductivity, and specific surface area. For instance, the SHAP analysis framework could help to identify key factors affecting mechanical properties, while the prediction model could guide the design of aerogels with tailored porosity and density. This extensibility makes our approach a valuable tool for comprehensive aerogel material design.

## Data Availability

The data presented in this study are available on request from the corresponding authors.

## Supplementary Materials

The following supporting information can be downloaded at: https://www.mdpi.com/article/10.3390/gels11010070/s1, Table S1: Statistical analysis of features in the dataset, Data S2: Literature Data Sources.
