# Supplementary material for: Machine Learning Models for Predicting Thermal Properties of Radiative Cooling Aerogels
Source: Gels. 2025 Jan 16;11(1):70. doi: 10.3390/gels11010070 (PMC11765191; doi:10.3390/gels11010070)
Supplement: Supplementary file 1 [file gels-11-00070-s001.zip › gels-3397331-supplementary.pdf]

Supporting Information

Contents

1. Dataset Details

2. Literature Data Sources

1. Dataset Details

Table S1. Statistical analysis of features in the dataset

	ratio_num(wt%)	modifier_content(wt%)	SR(%)	MIR(W/m²)	SOLAR	Air(°C)	T(°C)
Mean	90.82	0.15	0.43	92.17	477.59	32.30	8.72
Std	15.94	0.15	4.54	4.21	220.86	10.44	4.83
Min	55.00	0.00	78.00	80.00	0.00	5.48	0.89
25%	82.00	0.00	89.00	90.00	285.14	24.81	4.56
50%	100.00	0.04	92.00	92.00	477.59	31.61	8.72
75%	100.00	v	95.00	94.00	612.71	41.20	11.74
Max	100.00	0.43	98.00	98.00	912.34	57.36	40.28

The continuous variables (ratio_num, modifier_content, SR, MIR, SOLAR, air, and T) are presented with their statistical values in the table above. For the matrix materials and modifiers, one-hot encoding was used where:

- Matrix materials (CNF, CS, CaSiO3, MTMS, PAAm, PDMS, PEA, PLA, PPU, PVA, PVDF, SA) are encoded as binary variables (0 or 1)

- Modifiers (1,4-Dioxane, Al2O3, AlCl, CaCO3, LiCl, PDA, PTFE, SiO2, TiO2, WA, ZnO, ZrO2) are encoded as binary variables (0 or 1)

When a specific material or modifier is present in a sample, its corresponding variable is set to 1; otherwise, it is set to 0.

Data S2. Literature Data Sources

The experimental data used in this study were extracted from the following published literature. These papers constitute the primary data sources for our machine learning analysis:

1. Gu, B.; Fan, F.; Xu, Q.; Shou, D.; Zhao, D. A Nano-Structured Bilayer Asymmetric Wettability Textile for Efficient Personal Thermal and Moisture Management in High-Temperature Environments. *Chemical Engineering Journal* **2023**, *461*, 141919, doi:10.1016/j.cej.2023.141919.

2. Shan, X.; Liu, L.; Wu, Y.; Yuan, D.; Wang, J.; Zhang, C.; Wang, J. Aerogel-Functionalized Thermoplastic Polyurethane as Waterproof, Breathable Freestanding Films and Coatings for Passive Daytime Radiative Cooling. *Advanced Science* **2022**, *9*, 2201190, doi:10.1002/advs.202201190.

3. Li, T.; Sun, H.; Yang, M.; Zhang, C.; Lv, S.; Li, B.; Chen, L.; Sun, D. All-Ceramic, Compressible and Scalable Nanofibrous Aerogels for Subambient Daytime Radiative Cooling. *Chemical Engineering Journal* **2023**, *452*, 139518, doi:10.1016/j.cej.2022.139518.

4. Cai, W.; Lin, B.; Qi, L.; Cui, T.; Li, Z.; Wang, J.; Li, S.; Cao, C.; Ziaur Rahman, M.; Hu, X.; et al. Bio-Based and Fireproof Radiative Cooling Aerogel Film: Achieving Higher Sustainability and Safety. *Chemical Engineering Journal* **2024**, *488*, 150784, doi:10.1016/j.cej.2024.150784.

5. Cai, C.; Chen, W.; Wei, Z.; Ding, C.; Sun, B.; Gerhard, C.; Fu, Y.; Zhang, K. Bioinspired “Aerogel Grating” with Metasurfaces for Durable Daytime Radiative Cooling for Year-Round Energy Savings. *Nano Energy* **2023**, *114*, 108625, doi:10.1016/j.nanoen.2023.108625.

6. Shi, M.; Song, Z.; Ni, J.; Du, X.; Cao, Y.; Yang, Y.; Wang, W.; Wang, J. Dual-Mode Porous Polymeric Films with Coral-like Hierarchical Structure for All-Day Radiative Cooling and Heating. *ACS Nano* **2023**, *17*, 2029–2038, doi:10.1021/acsnano.2c07293.

7. Yang, W.; Xiao, P.; Li, S.; Deng, F.; Ni, F.; Zhang, C.; Gu, J.; Yang, J.; Kuo, S.-W.; Geng, F.; et al. Engineering Structural Janus MXene-Nanofibrils Aerogels for

Season-Adaptive Radiative Thermal Regulation. *Small* **2023**, *19*, 2302509, doi:10.1002/smll.202302509.

8. Chen, Y.; Sun, Y.; Cheng, F.; Ji, Y.; Cai, C.; Fu, Y. Harmonizing Sunlight and Architecture for Argema-Inspired Nanocellulose Aerogel Radiative Coolers via Energy Tailoring Strategy. *ACS Sustainable Chem. Eng.* **2024**, *12*, 10680–10692, doi:10.1021/acssuschemeng.4c00300.

9. Zhong, S.; Yuan, S.; Zhang, X.; Zhang, J.; Xu, L.; Xu, T.; Zuo, T.; Cai, Y.; Yi, L. Hierarchical Cellulose Aerogel Reinforced with In Situ-Assembled Cellulose Nanofibers for Building Cooling. *ACS Appl. Mater. Interfaces* **2023**, *15*, 39807–39817, doi:10.1021/acsami.3c06178.

10. Lan, P.-H.; Hwang, C.-W.; Chen, T.-C.; Wang, T.-W.; Chen, H.-L.; Wan, D. Hierarchical Ceramic Nanofibrous Aerogels for Universal Passive Radiative Cooling. *Advanced Functional Materials* **2024**, *34*, 2410285, doi:10.1002/adfm.202410285.

11. Liu, X.; Zhang, M.; Hou, Y.; Pan, Y.; Liu, C.; Shen, C. Hierarchically Superhydrophobic Stereo-Complex Poly (Lactic Acid) Aerogel for Daytime Radiative Cooling. *Advanced Functional Materials* **2022**, *32*, 2207414, doi:10.1002/adfm.202207414.

12. Leroy, A.; Bhatia, B.; Kelsall, C.C.; Castillejo-Cuberos, A.; Di Capua H., M.; Zhao, L.; Zhang, L.; Guzman, A.M.; Wang, E.N. High-Performance Subambient Radiative Cooling Enabled by Optically Selective and Thermally Insulating Polyethylene Aerogel. *Science Advances* **2019**, *5*, eaat9480, doi:10.1126/sciadv.aat9480.

13. Han, D.; Wang, C.; Han, C.B.; Cui, Y.; Ren, W.R.; Zhao, W.K.; Jiang, Q.; Yan, H. Highly Optically Selective and Thermally Insulating Porous Calcium Silicate Composite SiO2 Aerogel Coating for Daytime Radiative Cooling. *ACS Appl. Mater. Interfaces* **2024**, *16*, 9303–9312, doi:10.1021/acsami.3c18101.

14. Deng, C.; Zhang, H.; Zhao, B.; Chen, Y.; Li, Y.; Zhang, T.; Qiu, F. Highly Strength and Flame-Retardant Aerogel Cooler with Reticular Porous Structures for Building Energy Saving. *Solar Energy* **2024**, *279*, 112840, doi:10.1016/j.solener.2024.112840.

15. Yu, Y.; Wei, L.; Pang, Z.; Wu, J.; Dong, Y.; Pan, X.; Hu, J.; Qu, J.; Li, J.; Tian, D.; et al. Multifunctional Wood Composite Aerogel with Integrated Radiant Cooling and Fog–Water Harvesting for All-Day Building Energy Conservation. *Advanced Functional Materials n/a*, 2414590, doi:10.1002/adfm.202414590.

16. He, R.; Liao, Y.; Huang, J.; Cheng, T.; Zhang, X.; Yang, P.; Liu, H.; Liu, K. Radiant Air-Conditioning with Infrared Transparent Polyethylene Aerogel. *Materials Today Energy* **2021**, *21*, 100800, doi:10.1016/j.mtener.2021.100800.

17. Liu, Y.; Bu, X.; He, M.; Liang, S.; Zhou, Y. Robust Passive Daytime Radiative Coolers Based on Thermally Insulating and Spectrally Selective Composite

Aerogels with Designed Fiber-Reinforced Porous Architecture. *Solar Energy* **2022**, *247*, 564 – 573, doi:10.1016/j.solener.2022.10.063.

18. Ma, C.-Q.; Xue, C.-H.; Fan, W.; Guo, X.-J.; Cheng, J.; Huang, M.-C.; Wang, H.-D.; Wu, Y.-G.; Liu, B.-Y.; Lv, S.-Q. Synchronous Radiative Cooling and Thermal Insulation in SiO2/Poly(Vinyl Alcohol) Composite Aerogel for Energy Savings in Building Thermal Management. *ACS Sustainable Chem. Eng.* **2024**, *12*, 5695 – 5704, doi:10.1021/acssuschemeng.4c00773.

19. Fan, W.; Gao, Q.; Xiang, J.; Yan, J.; Chen, Y.; Fan, H. Synergistic Effect of Silica Aerogel and Titanium Dioxide in Porous Polyurethane Composite Coating with Enhanced Passive Radiative Cooling Performance. *Progress in Organic Coatings* **2023**, *183*, 107763, doi:10.1016/j.porgcoat.2023.107763.

20. Cai, W.; Li, Z.; Xie, H.; Wang, W.; Cui, T.; Lin, B.; Qi, L.; Hu, X.; Du, Y.; Ming, Y.; et al. Thermally Managed and Fireproof Composite Aerogels for Safer and Year-Round Energy Saving. *Chemical Engineering Journal* **2024**, *483*, 149006, doi:10.1016/j.cej.2024.149006.

.
